# Blue Laser Diode Based Free-space Optical Data Transmission elevated to 18 Gbps over 16 m

**DOI:** 10.1038/s41598-017-10289-y

**Published:** 2017-09-05

**Authors:** Yu-Fang Huang, Yu-Chieh Chi, Hsuan-Yun Kao, Chen-Ting Tsai, Huai-Yung Wang, Hao-Chung Kuo, Shuji Nakamura, Ding-Wei Huang, Gong-Ru Lin

**Affiliations:** 1 Graduate Institute of Photonics and Optoelectronics, and the Department of Electrical Engineering, National Taiwan University, No. 1, Sec. 4, Roosevelt Road, Taipei, 10617 R.O.C. Taiwan; 2 Deaprtment of Photonics, National Chiao Tung University, 1001 Ta Hsueh Rd., Hsinchu, 30050 R.O.C. Taiwan; 3 Materials Department, University of California, Santa Barbara, Santa Barbara, CA 93106-5050 USA

## Abstract

Up to 18-Gbps direct encoding of blue laser diode (BLD) is demonstrated for free-space data transmission. By reshaping the orthogonal frequency multiplexed (16-QAM OFDM) stream with sidelobe filtering, the raw data rate expedites from 17.2 to 18.4 Gbps. Employing an ultrafast p-i-n photodiode with smaller active area diameter and lower noise equivalent power significantly enlarges the data rate by 1.6 Gbps or upgrades the signal-to-noise ratio (SNR) by 0.2 dB. Replacing the 80-mW BLD with the 120-mW one essentially increases the received SNR by 0.4 dB under enhanced modulation throughput. Reinforcing the beam collimation and collection by increasing the numerical aperture with a plano-convex hyper-hemispherical lens further improves the SNR by 0.6 dB. After optimization, the 16-QAM OFDM data with and without sidelobe filtering are respectively delivered at raw data rates of 16.4 and 18 Gbps with spectral-density usage efficiency as high as 4 bit/s/Hz over 16 m in free space, wherein the BLD carried QAM-OFDM data stream remains its capacity after reformation with sidelobe filtering as the superior inter-carrier-interference immunity reinforces.

## Introduction

The ardently developed optical wireless communication (OWC) with well-known advantages of electromagnetic interference immunity and confidential flexibility as compared to typical Wi-Fi, has emerged as suitable alternative for local area networks, in aircraft cabin and hospital environments^1^. With the rapidly increased capacity demand of mobile communication, visible light carrier covering wavelength of 380–780 nm efficiently assists the data communication to relieve the foreseeable network congestion in the near future. As the Light Fidelity (Li-Fi) named by Haas in 2011^2^, some Li-Fi prototypes based on light emitting diode (LED) carriers have been realized in retail stores, hypermarkets, hospitals and even driverless cars. Versatile services including lighting communication link with camera for positioning guides, incessant monitor and medical equipment access can be provided. Recently, the data transmission with a phosphorescent white LED using rate-adaptive discrete multi-tone modulation at 1 Gbps is proposed^3^, and a red/green/blue (RGB) white LED Li-Fi which supports 3.4-Gbps data-stream over 15 cm in free space is successfully launched^4^. Owing to the drawback of incoherence, the modulation bandwidth of typical LEDs is limited at several tens of MHz^5, 6^. In contrast, the laser diode (LD) with high coherence, super brightness and wide modulation bandwidth is considered as the alternative Li-Fi transmitter in next generation^7, 8^. The high-speed transmission with a LD/phosphor based white-light carrier can carry the rate-adaptive discrete multi-tone modulation up to 5.2 Gbps^9^. Alternatively, a red/green/blue (RGB) LD mixed white-light beam also launches the Li-Fi up to 8-Gbps over 50 cm in free space^10^. The white lighting functionality can be approached by either adhering yellow-phosphor to blue LD (BLD) or embedding tri-color RGB LDs in packages. Under such a vision of application, the ultimate transmission capacity of the sole BLD becomes a research spotlight at present. Not long ago, Watson *et al*. proposed a 2.5-Gbps visible light communication (VLC) system based on a 422-nm GaN LD with direct modulation of non-return-to-zero on-off-keying (NRZ-OOK) data in a 0.5-m free-space channel^11^. Afterwards, using a 450-nm GaN LD for 4-Gbps NRZ-OOK^12^ modulation over 0.15 m and a 410-nm LD for 5-Gbps NRZ-OOK modulation at back-to-back in the free-space environment are accomplished^13^. Remarkably, a high-speed VLC based on a GaN BLD for 9-Gbps orthogonal frequency division multiplexed 64-quadrature amplitude modulation (64-QAM OFDM) data over 5-m in free space is achieved by Chi *et al*. as a worldwide record to data^14^. Nevertheless, the free-space transmission capacity and distance of the BLD based VLC still have potential to be upgraded with improving the optical hardware and the data formats. In addition, the hemispherical plano-convex (P.C.) lens with large numerical aperture (NA) has never been employed for beam collimation and collection in any BLD based VLC system previously.

In this work, by improving the optical architecture with large-NA lens pair and ultrafast p-i-n photodiode (U-PD), together with the use of the 16-QAM OFDM data format without or with sidelobe filter, the BLD based 16-QAM OFDM communication link in free-space is demonstrated to enable point-to-point transmission up to 18 Gbps. To optimize the transmission capacity of the directly encoded BLD based VLC link in an orderly way, different scenarios of the BLDs, the lens pairs and the PD sets are selectable for implementing the system. The considered components for comparison include two BLDs with different output powers, two lens pairs with different focal lengths, apertures and NAs, and two p-i-n PDs with different bandwidths and noise equivalent powers (NEPs). With the use of large-NA P.C. lens for beam collimation, the U-PD successfully receives QAM-OFDM data stream with high signal-to-noise ratio (SNR), and the sidelobe filter effectively reduces the noises to increase the maximal allowable bandwidths to be transmitted.

## Results

**Figure 1:**
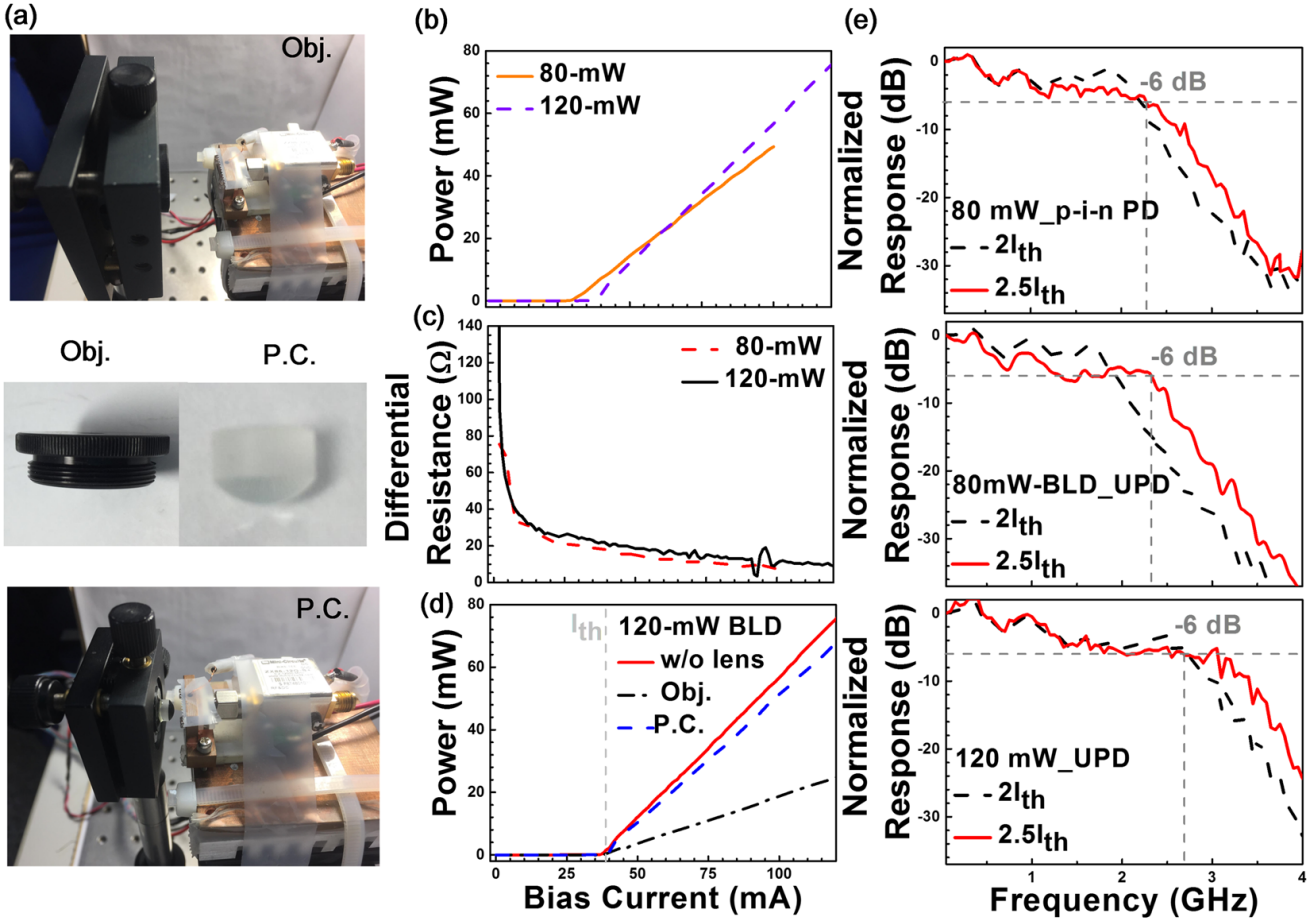
The pictures of BLD transmitter without and with Obj./P.C. lens. (**a**) The P-I and (**b**) differential resistance responses of 80-/120-mW BLDs. (**d**) The P-I curves of the 120-mW BLD combined without and with Obj./P.C. lens. (**e**) The frequency responses of 80-/120-mW BLD and p-i-n/U-PD sets.

Figure 1(a) shows the images of BLD transmitter with either objective (Obj.) or P.C. lens. Figure 1(b) depicts the power-to-current (P-I) responses of 80- and 120-mW BLDs with corresponding threshold currents are 30 and 35 mA, respectively. The output power for the 120-mW BLD is 43 mW, which is higher than that of 32 mW for the 80-mW one at same bias current ratio of 2.5I_th_. The 120-mW BLD with a higher P-I slope of 0.89 W/A provides higher modulation depth than the 80-mW BLD with a P-I slope of only 0.69 W/A. To investigate the return loss of used BLDs, the differential resistances of 80- and 120-mW BLDs are compared in Fig. 1(c). Note that the 80-mW BLD exhibits a differential resistance of 10.75 ohm with a return loss of −3.8 dB at an optimized current of 78 mA. In contrast, a similar differential resistance of 11 ohms is also obtained to give a return loss of −3.9 dB for the 120-mW BLD at an optimized current of 105 mA.

The Fig. 1(d) shows the P-I response of the 120-mW BLD combined without and with Obj./P.C. lens. With the use of P.C. lens, the P-I slope is slightly decreased from 0.89 W/A to 0.79 W/A, which is significantly higher than that of 0.3 W/A using the Obj. lens because of its smaller NA. The hyper-hemispherical P.C. lens effectively collimates the BLD beam to improve the transmission performance. During experiment, the frequency responses of 80-/120-mW BLDs and p-i-n/U-PDs sets before digital data encoding are analyzed. As a result, the frequency responses of the 80-mW BLD combined with p-i-n PD and U-PD are shown in Fig. 1(e). In principle, increasing the DC bias effectively up-shift the relaxation oscillation frequency to suppress the relative intensity noise (RIN) of the BLD. At the same bias current ratio of 2.5I_th_, the 80-mW BLD with p-i-n PD and U-PD exhibit similar −6 dB bandwidth of 2.3 GHz. Since the theoretical cut-off frequencies of the p-i-n-PD and U-PD are 3.3 GHz and >7 GHz, respectively, such a result indicates that the 80-mW BLD has already reached its maximal modulation bandwidth. In contrast, using the 120-mW BLD in combination with the U-PD reveals significantly broadened frequency response, which can provide a -6 dB modulation bandwidth of 3 GHz at a bias ratio of 2.5I_th_.

**Figure 2:**
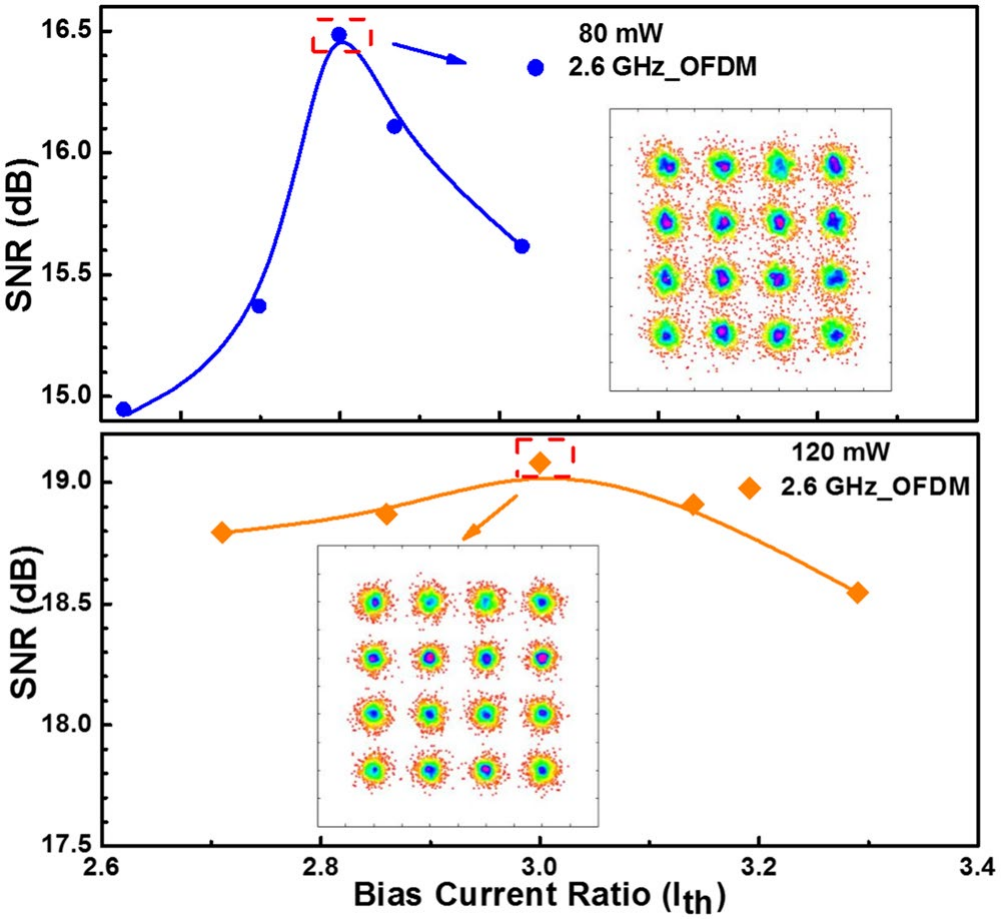
The subcarrier SNRs and related constellation plots of 0.5-m free-space transmitted 2.6-GHz 16-QAM OFDM data carried by the 80- and 120-mW BLDs at different bias current ratios.

**Figure 3:**
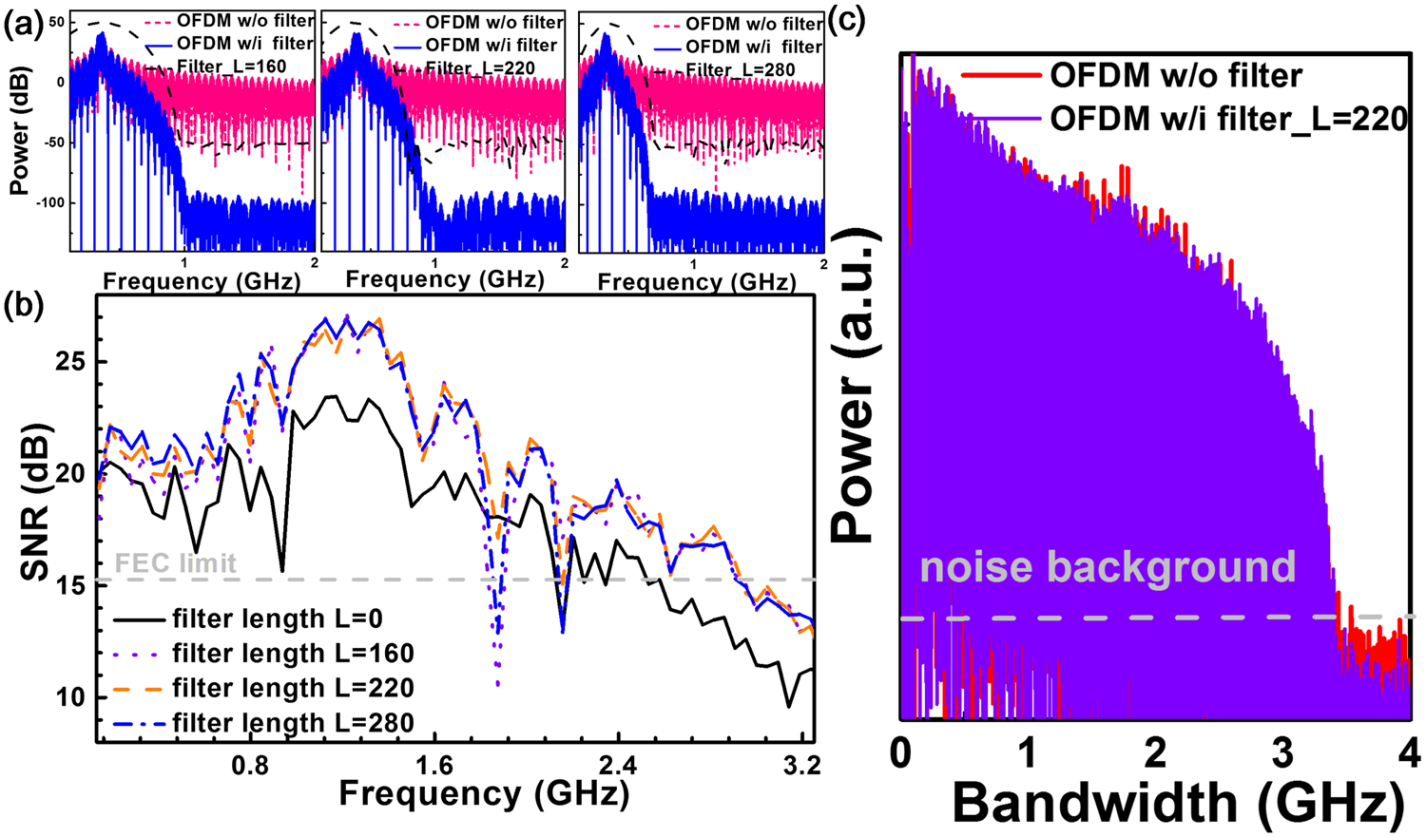
The use of OFDM with sidelobe filtering technique for suppressing the side lobe of OFDM subcarriers. (**a**) The RF spectra and (**b**) The subcarrier SNRs of 16-QAM OFDM data with sidelobe filter length of 0 (original OFDM), 160, 220 and 280, (**c**) The BtB transmitted 3.2-GHz 16-QAM OFDM data with sidelobe filter length of 0 and 220.

Later on, a 2.6-GHz-wide 16-QAM OFDM data is employed to directly encode two BLDs biased at different current ratios for optimization. To maintain the sufficiently broad modulation bandwidth at relatively low RIN with preserved modulation throughput and on/off extinction ratio, the optimized SNRs of 16.5 and 19.1 dB are observed at bias current ratios of 2.6I_th_ and 3I_th_ for the 80- and 120-mW BLDs, respectively, as shown in Fig. 2. Note that the SNR of the decoded QAM-OFDM can be obtained by using the EVM of the received constellation plot as described by refs 15 and 16:

1 $$SNR(n)\approx \frac{1}{EVM{(n)}^{2}}=\frac{{P}_{0}(n)}{{|{S}_{r}(n)-{S}_{t}(n)|}^{2}},$$

where *P*_0_ *(n)* denotes the average power of the QAM data carried by the nth OFDM subcarrier, *S*_*r*_ *(n)* and *S*_*t*_ *(n)* are the respective amplitudes of the received QAM data in a constellation point of the nth OFDM subcarrier. A sidelobe filter is added after each OFDM subcarrier to improve the data quality without suffering from the frequency and phase noises originated from the chirp of BLD. Figure 3(a) illustrates the RF spectra of one OFDM subcarrier with different window lengths (*Ls*) of sidelobe filter in time domain, in which the filter length of zero represents the original OFDM subcarrier. Note that the OFDM side lobes with CFO usually results in additional noise to degrade the received SNR performance.

In our experiments, the use of sidelobe filtering technique effectively suppresses the side lobe of OFDM subcarriers to optimize the SNR of the received QAM data after decoding. With a filter length of 160, the OFDM data spectrum significantly suppresses the carrier side lobes by 50 dB. Increasing the filter length to 280 further narrows the filtering window to suppress the low-harmonic-order side lobes. In detail, the SNR spectra of the 80-mW BLD carried 3.2-GHz 16-QAM OFDM data with different sidelobe filtering lengths are compared in Fig. 3(b). As compared to the typical OFDM data (filter length equivalent to 0), the 16-QAM OFDM data after FMBC filtering with a *L* of 160 significantly improves the average SNR from 15.6 to 17.5 dB, providing the SNR increment of 2 dB or beyond for all OFDM subcarriers. Lengthening the sidelobe filter length to 220 can further optimize the average SNR by 0.4 dB; however, overly increasing the filter length to 280 inversely degrades the average SNR even though the OFDM sidelobes can be greatly suppressed. Such a degradation results from the serious reshaping and distortion on the OFDM data spectrum with overly lengthened window of sidelobe filter in time domain. To reduce the degradation caused between the data and filter spectra during convolution, the sidelobe filter length should be properly selected for a compromise between the suppression of noise and the degradation of data. In addition to the overly filtering problem, each OFDM subcarrier also suffers from frequency and phase drifts due to the temporally varied chirp of the BLD, which causes additional noise on the carried data under the residual interference between carrier and sidelobes of adjacent carriers. The sidelobe filtering technique can be employed to suppress the sidelobe of each OFDM subcarrier. Such a filtering algorithm greatly reduces the sidelobes induced out-of-band noise among the OFDM subcarriers. This effectively improves the SNR of the received QAM-OFDM data. In our case, there is possibly a residual inter-carrier interference results from the carrier error induced by frequency chirp, phase noise and turn-on delay of the GaN BLD, and by the unsynchronized generator/receiver clock and the inequivalent sampling/resampling rate. The 0.5-m free-space transmitted electrical spectra of 3.2-GHz 16-QAM OFDM data without and with sidelobe filter (length of 220) carried by the BLD are shown in Fig. 3(c). Note that the sidelobe filtered QAM-OFDM data provides comparable power but exhibits lower noise background to indicate higher carrier-to-noise ratio (CNR) than the original OFDM data.

**Figure 4:**
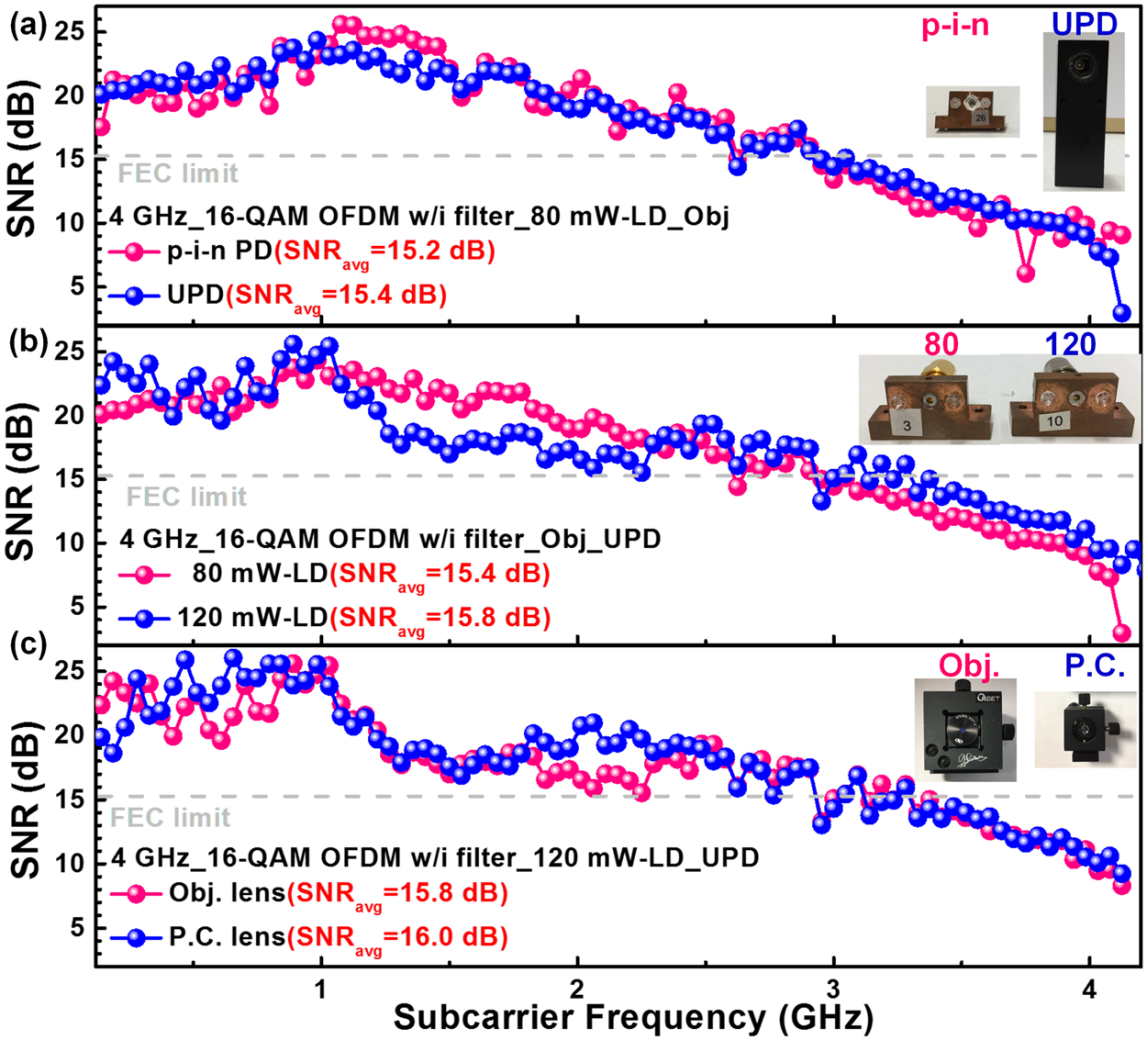
The step-by-step optimization with different sets of BLD, lens pair and PD for enhancing the VLC performance. The subcarrier SNRs of 16-QAM OFDM data with sidelobe filtering carried by the VLC system constructed by different combinations of (**a**) BLDs, (**b**) lens pairs and (**c**) PDs.

Subsequently, the step-by-step optimization with different sets of BLD, lens pair and PD are realized to enhance the VLC performance. For comparison, the 80-mW BLD delivered 16-Gbps 16-QAM sidelobe filtered OFDM data is received by either the p-i-n PD or the U-PD, as shown in Fig. 4(a). Although the U-PD is made with smaller aperture, its broader modulation bandwidth and lower NEP show better receiving performance than the p-i-n PD, which predominates the overall transmission performance to give better SNR of 15.4 dB and EVM of 17.1% for the 80-mW BLD case. The latter experiments are performed with the U-PD receiver. Later on, the subcarrier SNRs of the 4-GHz broadband 16-QAM OFDM data with sidelobe filtering carried by the 80- and 120-mW BLDs are shown in Fig. 4(b). Note that the 120-mW BLD can deliver the 16-Gbps data with respective SNR and EVM of 15.8 dB and 16.2% has shown better performances than those of 15.4 dB and 17.1% when carried by the 80-mW BLD, as the BLD with large power provide higher P-I slope to induce larger modulation depth and better throughput efficiency. Besides, the Obj. and P.C. lens pairs are compared in the VLC system at the same data rate, as shown in Fig. 4(c). Since the P.C. lens pair exhibits NA of 3-times larger than the Obj. lens pair, more BLD beam power can be collected to perform better SNR and EVM of 16 dB and 15.9%, respectively, at less systematic budget.

**Figure 5:**
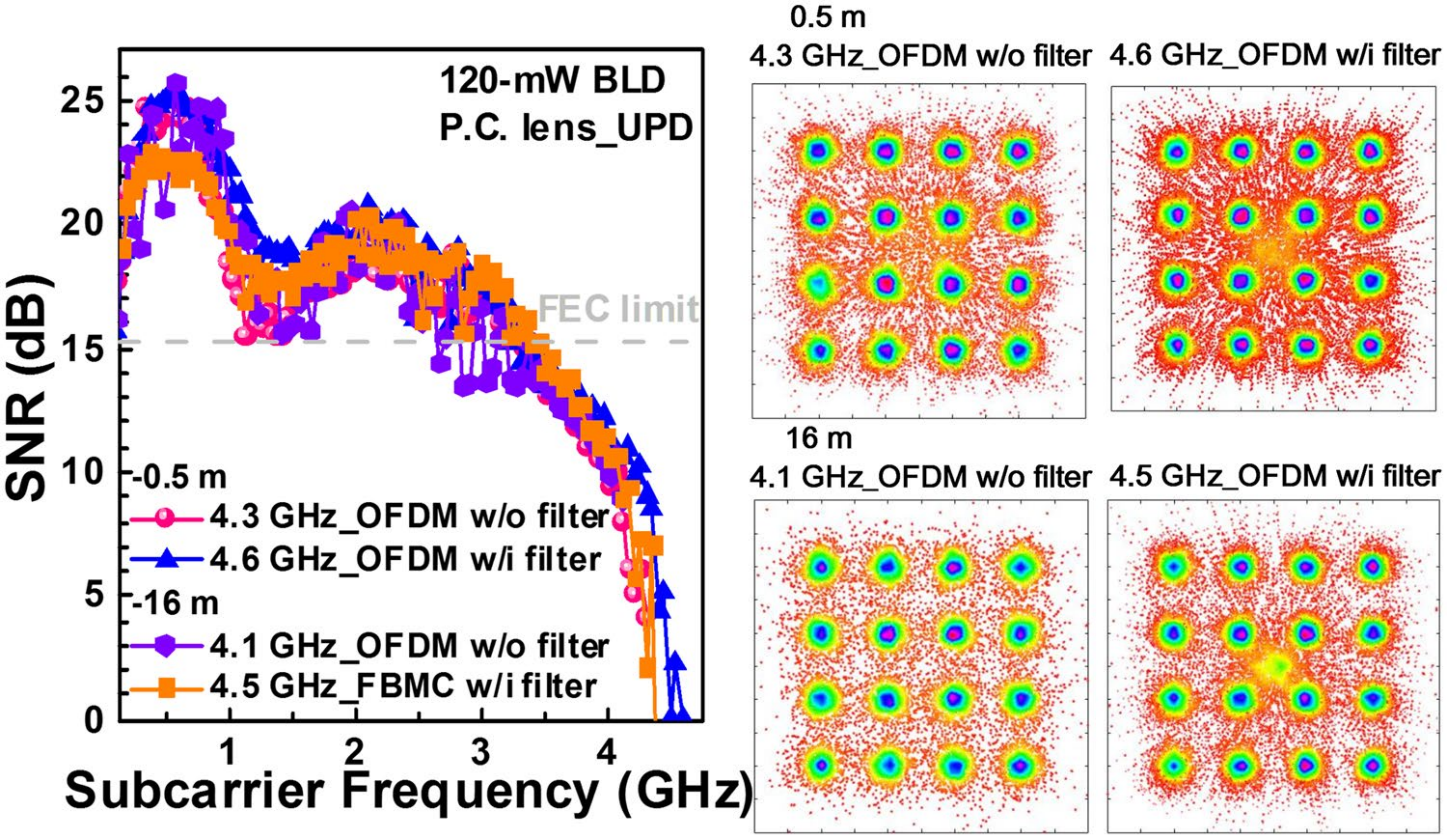
The subcarrier SNRs of the 16-QAM OFDM data without and with sidelobe filtering at maximal allowable data rates carried by the 80- and 120-mW BLDs after 0.5- and 16-m free-space transmissions.

Obviously, the combination of the 120-mW BLD, the P.C. lens pair and the U-PD can realize the VLC system with optimized data qualities. After the parametric optimization of components, the free-space transmission distance can lengthen to 16 m for the proposed VLC with the pre-leveling technique used for compensating the declined SNRs at high frequencies^17^. As a result from the related constellation plots, the subcarrier SNRs of the sidelobe filtered 16-QAM OFDM data surpass the SNR of 15.2 dB required by the FEC after 0.5- and 16-m free-space transmissions, as shown in Fig. 5. Therein the received optical powers after 0.5- and 16-m transmissions are 15200 and 12200 lux. With conventional OFDM format, the BLD based VLC enables the 16-QAM OFDM data transmission at raw data rates of 17.2 Gbps with 4.3-GHz bandwidth for 0.5-m distance and 16.4 Gbps with 4.1-GHz bandwidth for 16-m distance, providing average SNRs beyond 15.3 dB and EVM below 17%. Note that the maximal allowable OFDM data bandwidth is decreased by 0.2 GHz after 16-m free-space transmission with conventional OFDM format. In contrast, the use of the sidelobe filtering can even support a higher bandwidth of 4.6 GHz to provide a larger raw data rate of 18.4 Gbps. By employing the 120-mW BLD, the achievable transmission capacity a little degrades after propagating over 16 m in free space, which verifies the ability of the sidelobe filtering process on the inter-carrier-interference immunity to guarantee the transmission with lengthening distance. Note that the constellation point at origin of coordinate is mainly attributed to the significantly degraded SNRs at high subcarrier frequencies, which inevitably degrades the BER performance after off-line decoding by MATLAB problem.

**Figure 6:**
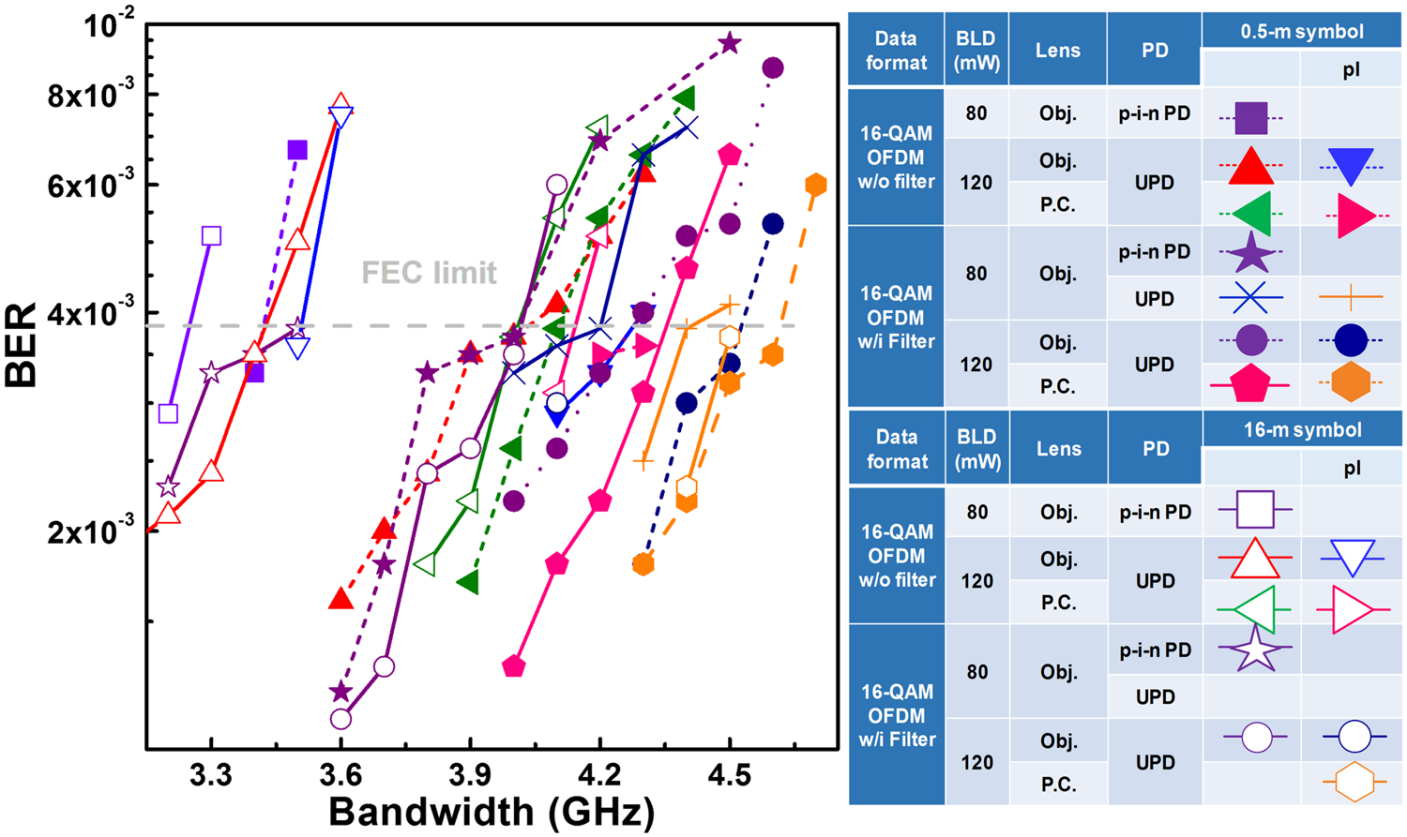
The BER performances of the BLD carried OFDM data without and with sidelobe filtering at different bandwidths over 0.5 and 16 m in free space.

In summary, the bit error rate (BER) performances of the 16-QAM OFDM data without or with sidelobe filter carried by the two BLDs with all kinds of components are compared in Fig. 6. The Obj. lens only allows the 80-mW BLD and the p-i-n PD set to support 13.6-/12.8-Gbps data rate after 0.5-/16-m free-space transmission. Replacing the transceiver with the 120-mW BLD and the U-PD not only enlarges the delivered power but also reduces the declined throughput of the QAM data carried by high-frequency OFDM subcarrier, which significantly enlarges its 0.5-/16-m transmission capacity to 16.8/15.2 Gbps after pre-leveling. Except the improvement on the P-I slope of the BLD and the responsivity of the U-PD, the lower NEP and the broader bandwidth are two important roles for improving the transmission performance. Changing the cost-ineffective Obj. lens by the larger-NA P.C. lens pair with equivalent focal length further improves the data capacities to 17.2 Gbps/0.5-m and 16.4 Gbps/16-m. Another essential enhancement arises from the use of sidelobe filtering on the OFDM data at the same QAM level, which greatly increases the deliverable raw data rates even with the earlier set of VLC with 80-mW LD, Obj. lens and p-i-n PD. By pre-leveling the sidelobe filtered OFDM subcarriers, the P.C.-lens collimated 120-mW BLD beam enlarges the 16-QAM OFDM transmission capacity up to 18.4 Gbps with a little degradation of 0.4 Gbps during 16-m free-space transmission, which is 4.5-Gbps larger than that with an 80-mW-BLD/Obj.-lens/p-i-n PD set.

Table 1 lists maximal allowable transmission capacities of the BLD based VLC system for 16-QAM OFDM without or with the sidelobe filtering. In previous work^14^, the achievable data rate of 9 Gbps for the 64-QAM OFDM data with occupied bandwidth of 1.5 GHz is performed by using the avalanche photodiode (APD) which can provide sufficiently high conversion gain and guarantee the received SNR to surpass the FEC criterion. However, the commercial APD exhibits a limited cut-off frequency bandwidth of only 900 MHz, which limits the proposed VLC system for carrying the 64-QAM OFDM data with bandwidth less than 1.5 GHz. To compare, the p-i-n PD with cascaded amplifier used in this work further improves the allowable bit-rate by almost 4 Gbps with a lengthened distance of 16 m. Alternatively, the high-power BLD and the broadband UPD further enlarge the transmission capacity by 6.2 Gbps. Furthermore, the increment can be up to 7.4 Gbps by employing the large-NA P.C. lens pair to take over the Obj. lens. The use of sidelobe filtered OFDM at the same QAM level further enlarges the transmission capacity by 7.4 Gbps with Obj. lens and by 9 Gbps with P.C. lens, which confirms its superiority on suppressing the BLD chirp induced noises to provide bandwidth enhancement and latency tolerance during distant transmission of such a BLD based VLC system.

**Table 1:** The maximal allowable transmission capacities of the BLD based VLC systems.

16-QAM Data	G_Amp1_ (dB)	BLD (mW)	Lens	PD	G_Amp2_ (dB)	Data rate (Gbps)	
						0.5 m	16 m
OFDM w/o filter	26	80	Obj.	p-i-n PD	18	13.6	12.8
		120	Obj.	UPD		16.8	15.2
			P.C.			17.2	16.4
OFDM w/i filter	26	80	Obj.	p-i-n PD	18	16	14
				UPD		17.6	
		120	Obj.	UPD		18	16.4
			P.C.			18.4	18

## Conclusion

In conclusion, by using a directly 16-QAM sidelobe filtered OFDM data to encode the BLD, the 18-Gbps VLC system with spectral-density usage efficiency as high as 4 bps/Hz for transmission over 16 m in free space is demonstrated. By comparing the transmission performances of the proposed VLC system constructed with different BLDs, lens pairs or PDs, the transmission capacity is optimized in sequence. At the same data rate, the use of BLD with high P-I slope, lens pair with large NA and PD with low NEP enables to demonstrate the VLC system with optimized transmission performances. In terms of detailed optimization, replacing the 80-mW BLD with the 120-mW one with P-I slope enlarging from 0.69 W/A to 0.89 W/A increases the received SNR by 0.4 dB. By employing the high power 120-mW BLD, it provides higher P-I slope to induce larger modulation depth and better throughput efficiency. In addition, increasing the NA of used lens pair from 0.25 to 0.75 improves the SNR by 0.6 dB. The transmission performs better SNR and EVM by collecting more BLD beam power. At the same responsivity, the U-PD with lower NEP but smaller active area diameter than the p-i-n PD still upgrades the SNR by 0.2 dB. Obviously, the performance enhancement relies on replacing the receiver with the smaller effective active area but broader modulation bandwidth and lower NEP U-PD than the conventional p-i-n PD, which predominates the overall transmission performance. On the other hand, for data format optimization, the OFDM data with sidelobe filtering greatly increases the deliverable raw data rates at the same QAM level. The properly selected filter length makes the sidelobe filtered OFDM data a higher SNR than the OFDM without filtering process. After optimization, the proposed VLC system at 0.5-m case enables to support 17.2- and 18.4-Gbps raw data rates on carrying 16-QAM OFDM without and with sidelobe filtering process, respectively, to pass the FEC criterion. After 16-m free-space transmission for the OFDM data, the transmission capacity of the VLC system is slightly decreased by 0.8 Gbps. By pre-leveling the sidelobe filtered OFDM subcarriers, the P.C.-lens collimated 120-mW BLD beam enlarges the 16-QAM OFDM transmission capacity up to 18 Gbps even with a little degradation of 0.4 Gbps during 16-m free-space transmission, which is much higher than the capacity of 13.6 Gbps provided by the 80-mW BLD/Obj.-lens/p-i-nPD set. At a distance of 0.5 m, the proposed VLC system enables to handle the 16-QAM sidelobe filtered OFDM data covering a bandwidth of 4.6 GHz for providing at a raw data rate of 18.4 Gbps, and a distinguish constellation plot with an EVM of 17.2%, the SNR of 15.3 dB and the BER of 3.5 × 10^−3^ are observed. Most important, the achievable transmission capacity remains unchanged even after lengthening the distance, which verifies the ability of the sidelobe filtering process on the superior noise immunity to guarantee the transmission with lengthening distance.

## Method

### The experimental setup of proposed VLC system for 16-QAM OFDM transmission over 16-m free space

**Figure 7:**
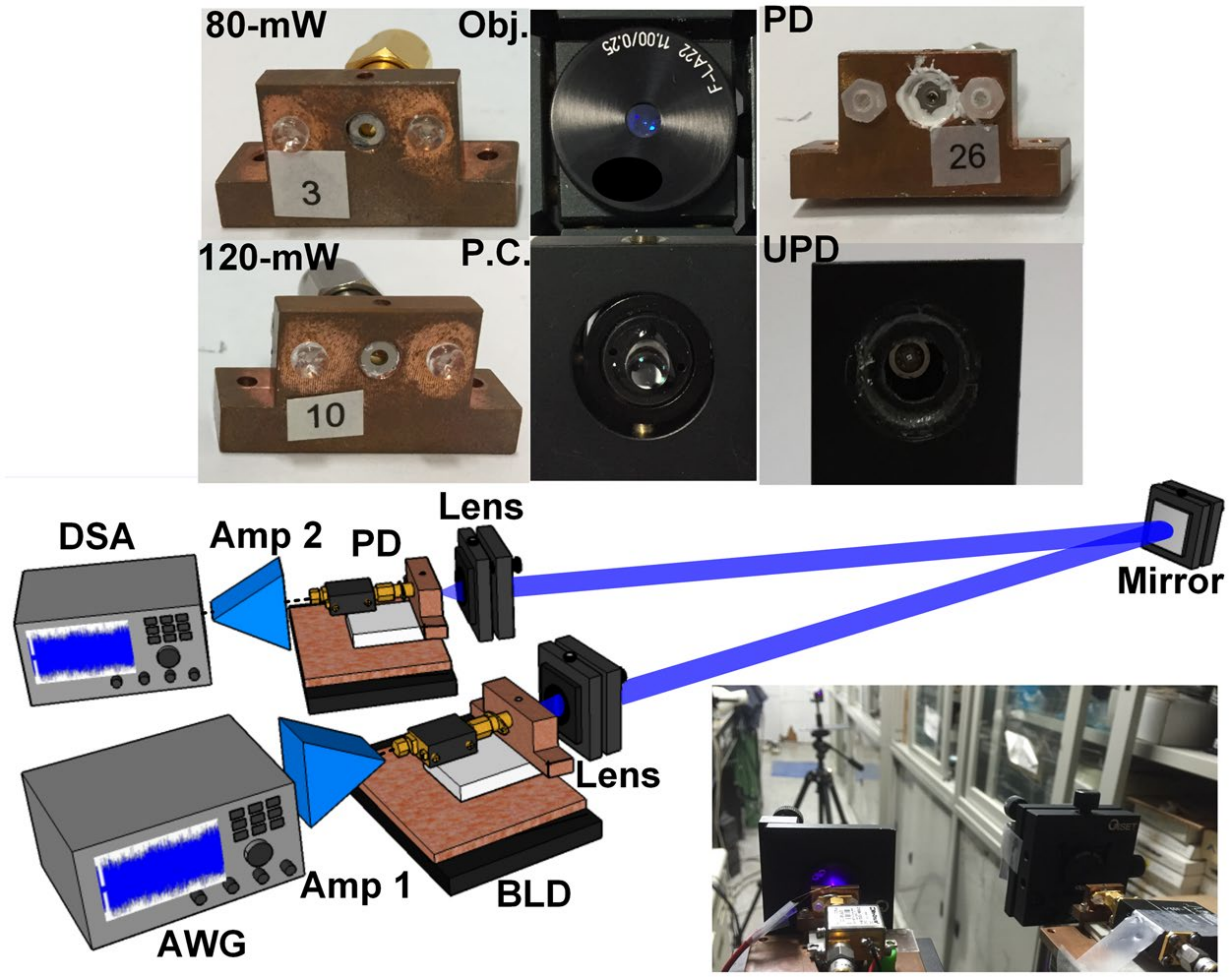
Images and experimental setup of the BLD based free-space data transmission.

The experimental setup of point-to-point (PtP) VLC system based on the commercially available TO-can packaged BLD over 16 m in free space is shown in Fig. 7. During experiments, two BLDs with maximal output powers of 80 (Osram, PL 450B) and 120 mW (Osram, PLT5 450B) are employed for comparison. In detail, the 80- and 120-mW BLDs with corresponding threshold currents of 30 and 35 mA exhibit power-to-current (P-I) slopes of 0.69 W/A and 0.89 W/A, respectively. The BLD is mounted upon the thermo-electric cooler connected with heat sink and controlled at room temperature of 25 °C for stabilizing their output dynamics. For data encoding, the 16-QAM OFDM data without or with sidelobe filtering generated from an arbitrary waveform generator (AWG, Tektronix 70001 A) with oversampling rate of 24 GS/s is amplified to a peak-to-peak voltage of 2.3 volts via a broadband preamplifier (Amp.1, Picosecond, 5865) with 26-dB gain (G_Amp.1_). To operate the BLD beyond threshold condition, a bias-tee (Mini-circuit, ZX85-12G-S+) is employed to combine the DC current and the amplified QAM-OFDM data for driving the BLD.

Two types of focusing lenses, including the aspherical Obj. and the hyper-hemispherical P.C., are compared for minimizing the laser beam divergence through collimation control. The Obj. and P.C. lenses with aperture diameters of 5.5 and 6 mm show NAs of 0.25 and 0.75, respectively. By using a reflective mirror (Thorlabs, BBSQ1-E02) with a reflectivity of >99%, the BLD beam that carried the OFDM data is launched into an 8-m free-space link and folded back via the mirror for one round-trip transmission to provide a total distance of 16 m. At receiving end, the optical data is received by a Si p-i-n PD (Thorlabs, FDS025) with an active area diameter of 0.25 mm and an NEP of 9.29 × 10^−15^ W/(Hz)^0.5^ for providing a receiving bandwidth of 3.3 GHz. Alternatively, an U-PD (Alphalas, UPD-50-SP) with an active area diameter of 0.1 mm and an NEP 1.2 × 10^−15^ W/(Hz)^0.5^ can also be applied for providing a receiving bandwidth of >7 GHz. In addition, two PDs exhibit the same responsivity of 0.15 A/W at 450 nm. Afterwards, the received data experiences a low-noise amplification (Amp.2, New focus, 1422) with 18-dB gain (G_Amp.2_). Finally, the OFDM data without or with sidelobe filtering process is resampled by a digital serial analyzer (DSA, Tektronix, 71604 C) with a sampling rate of 100-GS/s, and then demodulated by a homemade MATLAB program for evaluating the EVM, SNR and BER.
